# Expansion Microscopy for Beginners: Visualizing Microtubules in Expanded Cultured HeLa Cells

**DOI:** 10.1002/cpns.96

**Published:** 2020-06-04

**Authors:** Chi Zhang, Jeong Seuk Kang, Shoh M. Asano, Ruixuan Gao, Edward S. Boyden

**Affiliations:** ^1^ Media Lab Massachusetts Institute of Technology (MIT) Cambridge Massachusetts; ^2^ McGovern Institute MIT Cambridge Massachusetts; ^3^ John A. Paulson School of Engineering and Applied Sciences Harvard University Cambridge Massachusetts; ^4^ Department of Biological Engineering MIT Cambridge Massachusetts; ^5^ Department of Brain and Cognitive Sciences MIT Cambridge Massachusetts; ^6^ Koch Institute for Cancer Research MIT Cambridge Massachusetts; ^7^ Current address, Internal Medicine Research Unit Pfizer Inc. Cambridge Massachusetts

**Keywords:** antibody, beginner, clearing, expansion microscopy, fluorescence microscopy, HeLa cells, hydrogel, imaging, immunocytochemistry, immunohistochemistry, microscopy, microtubules, super‐resolution microscopy

## Abstract

Expansion microscopy (ExM) is a technique that physically expands preserved cells and tissues before microscope imaging, so that conventional diffraction‐limited microscopes can perform nanoscale‐resolution imaging. In ExM, biomolecules or their markers are linked to a dense, swellable gel network synthesized throughout a specimen. Mechanical homogenization of the sample (e.g., by protease digestion) and the addition of water enable isotropic swelling of the gel, so that the relative positions of biomolecules are preserved. We previously presented ExM protocols for analyzing proteins and RNAs in cells and tissues. Here we describe a cookbook‐style ExM protocol for expanding cultured HeLa cells with immunostained microtubules, aimed to help newcomers familiarize themselves with the experimental setups and skills required to successfully perform ExM. Our aim is to help beginners, or students in a wet‐lab classroom setting, learn all the key steps of ExM. © 2020 The Authors.

## INTRODUCTION

The resolution of traditional optical microscopes is limited to a few hundred nanometers, a value set by the diffraction limit of light. As a result, nanoscale objects, such as immunostained microtubule filaments (estimated filament width ∼60 nm), are not clearly resolved when imaged using conventional, diffraction‐limited microscopes. Expansion microscopy (ExM) is an emerging technology that uses common lab equipment and commercially available, inexpensive chemicals to physically magnify biological specimens so that they can be imaged with nanoscale resolution under conventional, diffraction‐limited microscopes. During ExM, a swellable polymer network (or hydrogel) is uniformly synthesized throughout a biological specimen (with polymer threads winding their way around and between biomolecules, throughout and around cells). Biomolecules of interest (e.g., proteins, RNAs, and/or their markers) are anchored to the polymer network using commercially available crosslinking chemicals. Then, specimens undergo a process of softening, or mechanical homogenization (e.g., by proteolysis, or denaturation), followed by a process of isotropic three‐dimensional physical expansion when water is added, causing the polymer, and thus the specimen in which the polymer is embedded, to swell. As a result, biomolecules of interest are spatially separated from each other, and the effective resolution of the microscope is increased. For a recent review of the history of the field, with a broad survey of ExM methods and examples of applications to different parts of biology and medicine, please see Wassie, Zhao, & Boyden (2019).

After a typical ∼4.5× linear expansion (∼100× volumetric expansion) of a specimen, the effective resolution of a microscope objective lens with ∼300‐nm diffraction‐limited resolution becomes ∼70 nm (300 nm divided by ∼4.5). Since the basic concept behind ExM was established a few years ago (Chen, Tillberg, & Boyden, 2015), many different versions of ExM have been introduced by our and other labs. To name a few, protein‐retention forms of expansion microscopy (Chozinski et al., 2016; Gambarotto et al., 2019; Ku et al., 2016; Tillberg et al., 2016; Truckenbrodt et al., 2018) enable the expansion of cells and tissues labeled with standard fluorescent proteins and antibodies; expansion fluorescence in situ hybridization (ExFISH; Chen et al., 2016) allows the expansion and visualization of both proteins and RNAs; iterative expansion microscopy (iExM; Chang et al., 2017) allows repetitive expansion of the specimen through multiple rounds of polymerization and expansion (e.g., two rounds of expansion results in ∼4.5 × 4.5, or ∼20×, physical magnification); expansion pathology (ExPath) optimizes ExM for preserved human specimens (Zhao et al., 2017); and lattice light‐sheet microscopy applied to ExM‐processed specimens (Gao et al., 2019) enables fast imaging with nanoscale resolution and molecular contrast across large volumes.

We earlier described best‐practice, step‐by‐step ExM protocols for proExM and ExFISH for both cell culture and tissue specimens, as well as detailed procedures for handling and mounting expanded samples and for imaging them with confocal and light‐sheet microscopes (Asano et al., 2018). To complement these previous protocols, we here present a beginner‐friendly cookbook‐style protocol for expanding HeLa cells with immunostained microtubules using proExM. The idea here was to create a tutorial exercise that newcomers to ExM can try out, and that is simple enough to be practiced on one's own but comprehensive enough that all the core skills and practices of ExM will be explored in the process of completing the exercise. This exercise may be useful in wet‐lab classroom settings, and indeed we have used it in workshop settings for cohorts of a few dozen students, as it allows students to make rapid progress, even as they learn all the core steps of ExM and acquire hands‐on practice.

We chose to expand HeLa cells with immunostained microtubules in this exercise because microtubules are widely used as model cellular structures for super‐resolution microscopy, the improved resolution enabled by ExM can be immediately appreciated by a beginner performing the protocol, and there are established cell fixation methods to preserve microtubule structures for super‐resolution imaging (Bates, Huang, Dempsey, & Zhuang, 2007), well‐validated commercial antibodies for tubulins, and successful examples and expected outcomes for proExM of cultured cells with microtubules labeled (Tillberg et al., 2016). As befits the tutorial/exercise nature of this protocol, we choose to utilize cultured HeLa cells as the biological specimens because they are readily available from commercial sources (see the Basic Protocol), relatively easy to culture and maintain, and highly amenable to antibody staining after fixation and permeabilization.

**Figure 1:**
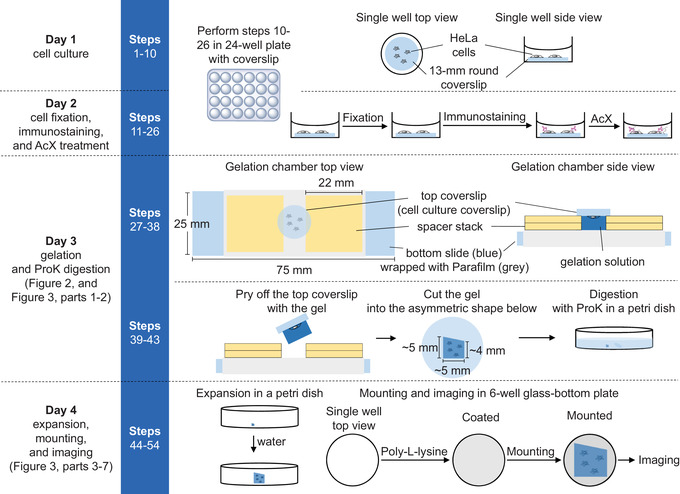
Key steps in expanding fixed HeLa cells with immunostained microtubules. AcX, 6‐((acryloyl)amino)hexanoic acid, succinimidyl ester; ProK, proteinase K.

In this 4‐day protocol (Fig. 1), we first culture HeLa cells on round coverslips placed in 24‐well plates (Day 1), and then perform cell fixation, immunostaining, and protein anchoring (Day 2). Next, we subject the cells to gelation using an optimized chamber design to minimize the physical handling and transfer of the hydrogel‐embedded sample, and then use proteinase K digestion to soften, or mechanically homogenize, the hydrogel‐embedded cells (Day 3). Finally, we expand the sample by water dialysis, and then mount the sample on a poly‐L‐lysine‐coated glass plate before fluorescence microscope imaging (Day 4).

We have tested this protocol with many beginners, including in the context of a hands‐on ExM training workshop at a medical school, and all of the participants in the workshop, with no prior experience with ExM, were able to successfully reproduce the protocol. Thus, we recommend that users consider using this protocol to learn the essential experimental setups and skills of ExM before applying and optimizing other, more flexible but more advanced ExM protocols (Asano et al., 2018, and protocols posted at http://expansionmicroscopy.org) to analyze their biological systems of interest. At the time of the writing of the current paper, more than 150 experimental papers and preprints involving some form of ExM have been published, suggesting that the field is poised for rapid growth. We hope that this protocol paper will help beginners get going with nanoimaging, a previously equipment‐ and/or complexity‐rich enterprise, but one that can now be reduced to a simple set of chemical processing steps.

## VISUALIZATION OF MICROTUBULES IN HeLa CELLS BY EXPANSION MICROSCOPY

Key steps involved in the expansion microscopy of fixed and immunostained HeLa cells are shown in Figure 1, and step‐by‐step instructions are provided below. Note that all steps up to the gelation (step 31) are performed on cells cultured on 13‐mm‐diameter coverslips placed in wells of a 24‐well plate. All the measures described below are for one well of a 24‐well plate; the measures should be scaled up linearly when handling multiple wells. Because of the time‐sensitive nature of the gelation process (steps 29 and 30), we recommend that beginners perform this protocol working with no more than two wells simultaneously. Please refer to the Reagents and Solutions section for details on the preparation of reagents and solutions described in the protocol.

### Materials

Actively growing culture of HeLa cells (ATCC, cat. no. CCL‐2) in cell culture medium (see recipe) in T‐75 culture flask
Dulbecco's phosphate‐buffered saline (DPBS) without calcium and magnesium (Thermo Fisher, cat. no. 14190136; store at room temperature for up to 36 months from the date of manufacture)
Trypsin‐EDTA solution (Thermo Fisher, cat. no. 25300054; store 3‐ml aliquots at −20°C for up to 24 months from the date of manufacture)
Cytoskeleton extraction buffer (see recipe)
Microtubule fixation solution (see recipe)
Reduction solution (see recipe)
Quenching solution (see recipe)
Immunostaining media kit (Active Motif, cat. no. 15251), containing MAXblock blocking medium, MAXbind staining medium, and MAXwash washing medium (store media up to 6 months at 4°C)
Primary antibody (rabbit polyclonal antibody to β‐tubulin, Abcam, cat. no. ab6046; store per manufacturer's guidelines)
Secondary antibody (goat anti‐rabbit IgG (H+L) highly cross‐adsorbed secondary antibody, Alexa Fluor 546 conjugate, Thermo Fisher, cat. no. A11035; store per manufacturer's guidelines)
6‐((Acryloyl)amino)hexanoic acid, succinimidyl ester (AcX) stock solution (see recipe)
Phosphate‐buffered saline (PBS; Corning, cat. no. 21‐040‐CV)
Monomer solution ("Stock X"; see recipe for preparation and storage), pre‐chilled on ice before performing step 29
*N*,*N*,*N*′,*N*′‐Tetramethylethylenediamine (TEMED) stock solution (see recipe for preparation and storage), pre‐chilled on ice before performing step 29
Ammonium persulfate (APS) stock solution (see recipe for preparation and storage), pre‐chilled on ice before performing step 29
Deionized water
Digestion buffer (see recipe)
Proteinase K (ProK; NEB, cat. no. P8107S; store per manufacturer's guidelines)
Poly‐l‐lysine solution (0.1% (w/v), Sigma Aldrich, cat. no. P8920‐100ML; stored up to 6 months at room temperature)

Water bath, 37°C, for warming cell culture reagents and solutions
Humidified cell culture incubator, set at 37°C and 5% CO_2_
Bucket of ice
Biosafety cabinet
T‐75 cell culture flasks (Corning, cat. no. 430641)
Cell counter (e.g., Invitrogen Countess II automated cell counter) or hemocytometer (e.g., Bulldog Bio, cat. no. DHC‐N01)
Vortex mixer
Chemical fume hood, for cell fixation (steps 11‐14)
13‐mm‐diameter round coverslips (Thermo Fisher, cat. no. 174950)
24‐well cell culture plates (Greiner Bio‐one, cat. no. 662160)
Parafilm (VWR, cat. no. 52858‐000)
Kimwipes (VWR, cat. no. 21905‐026)
Fluorescence microscope
22 × 22–mm square no. 1.5 coverslips (Brain Research Lab, cat. no. 2222‐1.5)
25 × 75–mm rectangular plain glass slides (VWR, cat. no. 48300‐026)
Reverse‐action forceps (VWR, cat. no. 89259‐942)
Syringe needles (VWR, cat. no. BD‐305196)
Clean, used pipet tip box containing empty pipet tip‐holding insert
37°C incubator, for gelation
Razor blades (VWR, cat. no. 55411‐050)
100 × 20–mm petri dish (Corning, cat. no. 353003)
Orbital shaker
Size 1/2 paintbrush (Utrecht, cat. no. 09311‐5012)
22 × 50–mm rectangular no. 1.5 coverslips (Brain Research Lab, cat. no. 2250‐1.5)
Size 6 paintbrush (Utrecht, cat. no. 09013‐1006)
6‐well glass‐bottom plate (CellVis, cat. no. P06‐1.5H‐N)

### Day 1: Cell culture

*IMPORTANT NOTE*: Perform all cell culture steps in a biosafety cabinet to ensure sterility. Prewarm cell culture medium, trypsin‐EDTA solution, and DPBS (without calcium and magnesium) to 37°C before cell culture experiments. The cells need to be passaged or plated when a plate reaches ∼70% confluency. A detailed mammalian cell culture protocol can be found in Phelan (2007).

The estimated experimental time on Day 1 is 2 hr.

### Passaging HeLa cells

1 Aspirate the cell culture medium from a T‐75 flask containing growing HeLa cells.
2 Add 2 ml prewarmed DPBS (without calcium and magnesium) to briefly rinse the cells, then aspirate the DPBS solution to remove all traces of cell culture medium (which contains trypsin inhibitors).
3 Add 2 ml prewarmed trypsin‐EDTA solution to the flask and incubate the flask for 5 min in the 37°C, 5% CO_2_ incubator, so that the cells detach from the flask surface.
  To avoid clumping, do not agitate the cells by hitting or shaking the flask while waiting for the cells to detach.
4 Add 8 ml prewarmed cell culture medium to the flask; gently pipet the solution up and down five times, ∼7 ml volume each time, to resuspend the detached cells in the cell culture medium.
5 To a fresh T‐75 flask, add 2 ml of the cell solution from step 4 and 8 ml prewarmed cell culture medium; gently pipet the solution up and down five times, ∼7 ml volume each time.
6 Incubate the HeLa cells in the T‐75 flask in the 37°C, 5% CO_2_ incubator until the next passage or plating.

### Plating HeLa cells on coverslips

7 Detach cells from the T‐75 flask by following steps 1‐4 of the protocol for passaging HeLa cells.
8 Determine the cell concentration using a cell counter (e.g., Invitrogen Countess II automated cell counter) following the manufacturer's protocol, or a hemocytometer (e.g., Bulldog Bio, cat. no. DHC‐N01) following Figure A.3B.1 and related steps in Phelan (2007).
9 Dilute the cells with prewarmed cell culture medium to a concentration of 50,000 cells/ml.
10 One day before you plan to begin step 11, insert a 13‐mm‐diameter coverslip into one well of a 24‐well plate and add 1 ml of the diluted cell solution. Incubate the plate in a humidified, 5% CO_2_ incubator at 37°C.
  The cells in the 24‐well plate will reach ∼70% confluency on Day 2 of the protocol.

### Day 2: Cell fixation, immunostaining, and AcX treatment

The following cell fixation and immunostaining steps are optimized for microtubules in HeLa cells and may not be applicable to other samples. See the introduction for references to other protocols for fixation and immunostaining of other types of specimens.

The estimated experimental time on Day 2 is 8 hr.

11 Aspirate the cell culture medium, and then add 500 µl cytoskeleton extraction buffer and incubate 1 min at room temperature.
12 Aspirate the cytoskeleton extraction buffer, add 1 ml microtubule fixation solution, and incubate 10 min at room temperature.
13 Aspirate the microtubule fixation solution, add 1 ml reduction solution, and incubate 7 min at room temperature.
14 Aspirate the reduction solution, add 1 ml quenching solution, and incubate 10 min at room temperature.
15 Aspirate the quenching solution, add 300 µl blocking medium (MAXblock blocking medium), and incubate 2 hr at room temperature.
16 Aspirate the blocking medium, add 1 ml washing medium (MAXwash washing medium), and incubate 10 min at room temperature.
  - During this incubation, prepare the primary antibody solution by diluting 3 µl of primary antibody with 297 µl of staining medium (MAXbind staining medium) in a 1.5‐ml microcentrifuge tube (1:100 dilution) and mixing the solution by pipetting up and down five times, ∼300 μl volume each time.
17 Aspirate the washing medium, add the primary antibody solution, and incubate 2 hr at room temperature.
18 Aspirate the primary antibody solution, add 1 ml washing medium, and incubate 10 min at room temperature.
19 Aspirate the washing medium, add 1 ml washing medium, and incubate 10 min at room temperature.
20 Repeat step 19 for an additional two wash cycles.
  - During the last period of washing, prepare the secondary antibody solution by diluting 3 µl secondary antibody with 297 µl staining medium in a 1.5‐ml microcentrifuge tube (1:100 dilution) and mixing the solution by pipetting up and down five times, ∼300 µl of volume each time.
21 Aspirate the washing medium, add the diluted secondary antibody solution, and incubate 2 hr at room temperature in the dark (e.g., by wrapping with aluminum foil).
22 Aspirate the secondary antibody solution, add 1 ml washing medium, and incubate 10 min at room temperature in the dark.
23 Aspirate the washing medium, add 1 ml fresh washing medium, and incubate 10 min at room temperature in the dark.
24 Repeat step 23 for an additional two wash cycles.
  - Before proceeding to step 25, check the cell fixation and immunostaining outcome by imaging the sample using a fluorescence microscope. The successfully fixed and immunostained microtubules should show as bright, continuous, but blurry lines with low background fluorescence (an example is shown at the end of the protocol).
25 Aspirate the washing medium, add 1 ml PBS, and incubate 5 min at room temperature in the dark.
  - During this 5‐min incubation, dilute the AcX stock solution in PBS as follows: add 3 µl AcX stock solution to 297 µl PBS for a total volume of 300 µl (1:100 dilution) in a microcentrifuge tube, and mix the solution by pipetting up and down five times, ∼300 µl volume each time.
26 Aspirate the PBS, add the diluted AcX solution, and incubate overnight (>6 hr) at room temperature in the dark.

### Day 3: Gelation and proteinase K digestion

The estimated experimental time on day 3 is 3 hr.

**Figure 2:**
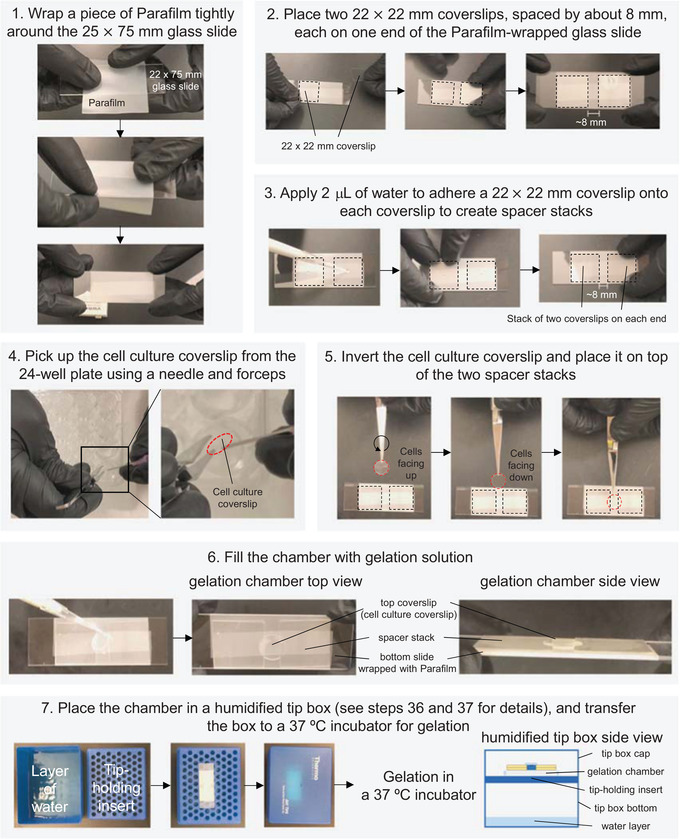
Construction of the gelation chamber and humidified tip box (protocol steps 31‐37).

27 Aspirate the diluted AcX solution from the 24‐well plate, add 1 ml PBS, and incubate 5 min at room temperature in the dark.
28 Aspirate the PBS solution, add 1 ml fresh PBS, and then place the 24‐well plate, wrapped with aluminum foil, on ice.
29 Prepare gelation solution by mixing Stock X, TEMED, and APS stock solutions in a 98:1:1 volumetric ratio.
  - All solutions should be chilled on ice before mixing.
  - For 1 ml gelation solution, mix 980 µl Stock X, 10 µl TEMED, and 10 µl APS stock solutions. APS must be added last.
  - Mix the solution by vortexing for 10 s, and then immediately place the solution back on ice to avoid premature polymerization. This 1 ml of gelation solution is enough for three wells of cells.
    This solution must be used immediately in step 30 after preparation. The final concentrations of chemicals in the gelation solution are 8.6% (w/v) sodium acrylate, 2.5% (w/v) acrylamide, 0.15% (w/v) N,N′‐methylenebisacrylamide, 2 M sodium chloride, 0.1% (w/v) TEMED, and 0.1% (w/v) APS, in PBS. Note that the TEMED and APS concentrations are half of those in our standard protocol (Asano et al., 2018), which allows more handling time for beginners, and avoids premature gelation.
30 Aspirate PBS from the 24‐well plate, add 300 µl gelation solution, and incubate the plate for 5 min on ice.
31 During the 5‐min incubation in step 30, prepare one gelation chamber for each well, as shown in Figure 2.
  - Wrap a piece of Parafilm around the glass slide (the “bottom slide”), pressing the piece of Parafilm gently onto the slide so that it wraps tightly around the slide (Fig. 2, part 1).
  - Place a square no. 1.5 coverslip (22 mm × 22 mm; the “spacer”) at each end of the Parafilm‐wrapped glass slide (Fig. 2, part 2).
    The spacers should be placed so that the width of the chamber (the gap between the spacers) is ∼8 mm (smaller than the diameter of the 13‐mm cell culture coverslip), as shown in Figure 2. Place a ruler under the bottom slide to help determine the spacing between the two coverslips. The distance between the two spacers should be smaller than the diameter of the coverslip so that the coverslip can rest on top of the two spacer stacks (cells face down) to form the gelation chamber.
  - Add 2 µl water on top of each spacer (Fig. 2, part 3), and then place another square no. 1.5 coverslip on top of each spacer, so that now on each end of the slide there is a stack of two coverslips.
    The water helps the two spacers to adhere together to create a thicker spacer stack so that the gel formed will be thicker and easier to handle for beginners.
  - Gently press the spacer stack so that the entire stack adheres to the surface of the Parafilm, being careful not to press too hard, as the coverslips might break.
    The top and side profiles of the gelation chamber are shown in Figures 1 and 2. The length of the chamber should be ∼8 mm, and the height of the chamber should be ∼0.34 mm—that is, the thickness of a stack of two no. 1.5 coverslips.

**Figure 3:**
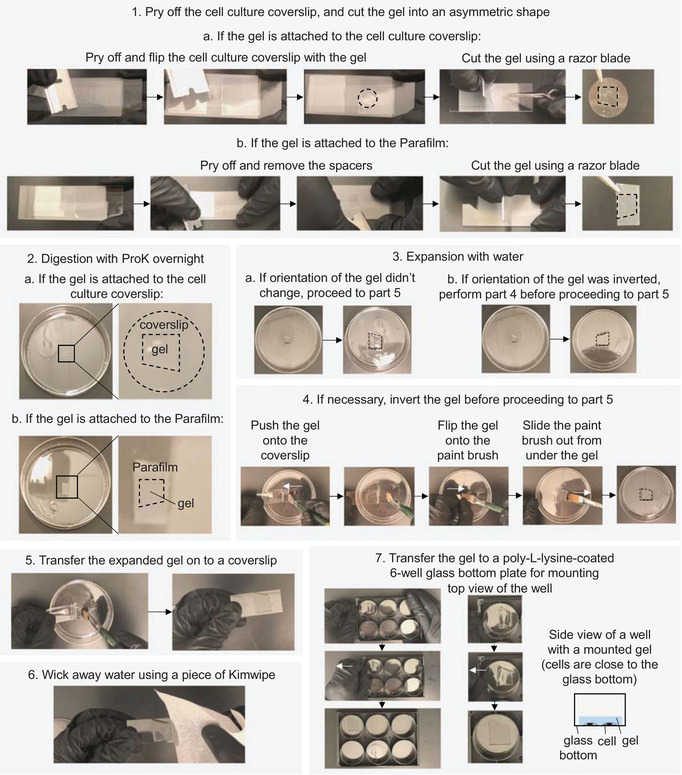
Digestion, expansion, and mounting of the sample (steps 40‐54).

32 After the 5‐min incubation in step 30, and the completion of chamber preparation from step 31, use a pair of forceps and a syringe needle with a bent tip to carefully pick up the cell culture coverslip from the 24‐well plate (Fig. 2, part 4). Proceed immediately to step 33 so that the cells do not dry out.
  For example, use a syringe needle with a bent tip to push the coverslip to the edge of the well, and then place the bent needle tip underneath the coverslip, lift the coverslip, and then pick up the coverslip using a pair of forceps. The 24‐well plate should be kept on ice during this step.
33 Invert the cell culture coverslip so that the cells face down, towards the Parafilm‐coated slide, and gently place it on top of the spacer stacks (Fig. 2, part 5). The edges of the coverslip will rest on the spacer stacks, with the cells suspended over the gap between the spacer stacks (see Fig. 1 for a profile view).
34 Use the forceps to gently press the cell culture coverslip down, so that it adheres (through surface tension) to the spacers.
35 Fill the space between the bottom slide, the spacers, and the inverted cell culture coverslip with 40 µl of gelation solution from the same well from which the cell culture coverslip came.
  - Carefully pipet out the gelation solution at the edge of the cell culture coverslip (Fig. 2, part 6), and it will be drawn under the coverslip through capillary force, effectively “filling” the chamber with gelation solution.
36 Add a layer of water to the bottom of a used, but clean, pipet tip box still containing its empty pipet tip‐holding insert. Then, place the chamber on the empty pipet tip‐holding insert (Fig. 2, part 7).
37 Close the tip box and transfer it into a 37°C incubator. Incubate 1 hr at 37°C, during which the gelation solution will polymerize.
  The gelation chamber is kept in a humidified environment so that the sample will not dry out during polymerization.
38 During the 1‐hr polymerization time in step 37, and before removing the gelled chamber from the incubator in step 39, prepare the digestion solution as follows: Mix proteinase K (ProK) with digestion buffer in a 1:100 volumetric ratio at room temperature. For each coverslip to be processed, prepare 10 ml ProK‐containing digestion buffer in a petri dish by mixing 10 ml of digestion buffer with 100 µl of ProK.
39 Remove the tip box from the 37°C incubator and let it cool to room temperature for 2 min. Take the gelation chamber out and set aside the tip box.
40 Slowly insert a razor blade from the side between the cell culture coverslip (the “top coverslip”) and the spacer stack to pry the cell culture coverslip off (Fig. 3, part 1).
  Because of the hydrophobicity of the Parafilm on the bottom slide, the gelled sample should be attached to the top coverslip (but if it is not, the gel can still be easily removed—see Figure 3, part 1b, and note at the end of step 43).

41 Use forceps to carefully flip the coverslip so that the gel is on top. Then, use the forceps to hold the cell culture coverslip by gently pressing it on the glass slide, and use a razor blade to trim the gelled sample on the top coverslip to approximately the asymmetric shape shown in Figure 1 and in Figure 3, part 1a.
  The dimensions of the asymmetric shape do not need to be precise, but the shape must be recognizable so that the orientation and spatial location of the cells in the gel can be easily determined.
42 Immerse the gelled sample and the accompanying coverslip, with the gel facing up and the coverslip at the bottom, in the previously prepared petri dish (step 38; Fig. 3, part 2) containing 10 ml of ProK‐containing digestion buffer.
43 Keep the sample on an orbital shaker at 60 rpm speed at room temperature overnight (>6 hr) in the dark (e.g., by wrapping the dish with aluminum foil).

Rarely, during step 40, the cell culture coverslip might be pried off while the gel remains attached to the Parafilm (Fig, 3, part 1b). In such cases, follow the steps below for sample processing.

- Pry off and remove the two spacer stacks, and then trim the gel, while on the Parafilm, using a razor blade into an asymmetric shape mirroring that shown in Figure 3, part 1a (see Figure 3, part 1b, for details).
- Use a razor blade to cut the Parafilm around the gel, and then transfer and immerse the gel together with the attached piece of Parafilm in the petri dish containing the ProK‐containing digestion solution, with the gel facing up and the Parafilm at the bottom.
- Keep the sample on an orbital shaker at 60 rpm at room temperature overnight (>6 hr) in the dark (e.g., by wrapping the dish with aluminum foil).

### Day 4: Expansion, mounting, and imaging

The estimated experimental time on day 4 is 3 hr.

**Figure 4:**
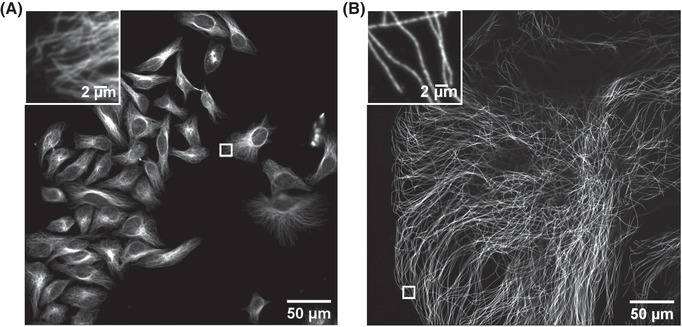
Imaging of HeLa cells with immunostained microtubules before and after expansion. Imaging was performed on an Andor spinning‐disk confocal microscope with a 40×, numerical aperture (NA) 1.15 water‐immersion objective. (**A**) Confocal image of HeLa cells with immunostained microtubules, imaged at a single *xy* plane at the bottom of the cells. The inset in the upper left zooms in on the small box at the middle right. (**B**) Confocal image of a ∼4.5× linearly expanded HeLa cell with immunostained microtubules, imaged at a single *xy* plane at the bottom of the cell. The inset in the upper left zooms in on the small box at the bottom left. Scale bars in (B) indicate post‐expansion scales.

44 After the digestion is complete, the cell culture coverslip (or the piece of Parafilm) should be detached from the gel, and the gel should expand ∼1.5 times linearly. Carefully aspirate the digestion buffer from the petri dish using a transfer pipet. Slightly tilt the petri dish under a lamp, if needed, to help locate the gel sample and thus avoid damaging the gel during the aspiration.
45 Carefully remove and discard the cell culture coverslip (or piece of Parafilm), using a pair of forceps.
46 After removing the digestion buffer and the cell culture coverslip (or piece of Parafilm), add 10 ml water to the dish (Fig. 3, part 3), and place the sample on the orbital shaker at 60 rpm for 10 min at room temperature in the dark (e.g., by wrapping the dish with aluminum foil).
  During each water‐expansion step, make sure that the gel is fully immersed in water so that the expansion process can proceed homogenously and quickly. If the gel floats on top of the water, use a wetted paintbrush to gently push the gel until submerged in the water.
47 Carefully aspirate the liquid in the petri dish containing the sample. Add 10 ml water, rewrap the petri dish with aluminum foil, and let the gel expand for 10 min at room temperature on the orbital shaker at 60 rpm.
48 Carefully aspirate the liquid in the petri dish containing the sample. Again add 10 ml water, rewrap the petri dish with aluminum foil, and let the gel expand for 20 min at room temperature on the orbital shaker at 60 rpm. The gel should now be fully expanded to ∼4.5× its original size.
  The gel is fully expanded when additional water treatment does not change the gel size. Inspect the shape of the gel after full expansion to determine which side the cells are on (using the asymmetric shape of the gel to ascertain which side was originally nearest to the coverslip, and thus contains the cells). If necessary, invert the gel by the following steps (Fig. 3, part 4).
  - *Carefully remove most, but not all, of the liquid from the petri dish*.
  - *Use one hand to hold and tilt a rectangular no. 1.5 coverslip (22 mm × 50 mm) next to the gel. Use a wetted size 6 paintbrush to push on the side of the gel and slide the gel onto the coverslip (Fig*. *3*, *part 4)*.
  - *Carefully tilt the coverslip and flip the gel onto the size 6 paintbrush, and then lower the paintbrush into the remaining liquid in the petri dish, and slide the paintbrush out from under the gel so that the gel is immersed in the liquid again, but inverted in orientation in relation to the petri dish*.
49 During the last water expansion, coat the 6‐well glass‐bottom plate with poly‐l‐lysine. Add 2 ml 0.1% (w/v) poly‐l‐lysine solution to each well, and incubate the plate on the orbital shaker for 10 min at 60 rpm at room temperature.
50 Aspirate the poly‐l‐lysine solution, add 2 ml water to each well, and incubate the plate 30 s at room temperature. Aspirate the water, and then dry the plate by blowing clean air or nitrogen over the glass‐bottom well surfaces. The plate is dry when there is no observable liquid.
51 Carefully remove as much liquid as possible from the petri dish containing the expanded gel sample.
52 Use a clean razor blade to cut the gel to a size smaller than 22 mm × 22 mm, if needed, maintaining an asymmetric shape so that the orientation can be easily assessed. Transfer the gel on to a coverslip by the following steps.
  - Use one hand to hold and tilt a rectangular no. 1.5 coverslip (22 mm × 50 mm) next to the gel.
  - Use a wetted size 6 paintbrush to push on the side of the gel and slide the gel onto the coverslip (Fig. 3, part 5).
  - Use a piece of a Kimwipe to wick away excess liquid (Fig. 3, part 6). Start absorbing excess liquid from the sides of the gel, and then carefully use the tip of the Kimwipe to absorb the liquid in between the bottom of the gel and the coverslip.
53 Slowly slide the gel into one well of the poly‐l‐lysine‐coated 6‐well glass bottom plate (Fig. 3, part 7) until the gel just contacts the poly‐l‐lysine surface, and then horizontally translate the slanted coverglass away from the contact site, to let the gel slide off from the coverslip and attach to the poly‐l‐lysine surface.
  Be careful not to introduce air bubbles between the bottom of the gel and the glass bottom of the 6‐well plate.
54 Carefully add a few drops of water to the top of the gel to keep it hydrated during imaging.
  The gel is now mounted and ready for imaging. In the example shown in Figure 3, part 7, the cells are closer to the glass bottom of the well, for imaging by an inverted microscope. The successfully expanded samples should show microtubule structures as bright, continuous sharp lines (see Fig. 4 for an example).

## REAGENTS AND SOLUTIONS

### AcX stock solution

**Table:** 

AcX stock solution	Amount	Final concentration
AcX (Thermo Fisher, cat. no. A20770)	5 mg	10 mg/ml
Anhydrous DMSO (Thermo Fisher, cat. no. D12345)	500 μl	
Total	500 μl	

The AcX stock solution can be divided into 10‐ to 20‐μl aliquots and stored up to 2 months at −20°C. The aliquots should be stored in a sealed container with drying agents (e.g., Drierite) or in a desiccator to avoid degradation by hydration.

### APS stock solution

**Table:** 

APS stock solution	Amount	Stock solution concentration (g/100 ml solution)
APS (Thermo Fisher, cat. no. 17874)	1 g	10
Water	9 ml	
Total	10 ml	

Divide APS stock solution into 1‐ml aliquots and store up to 2 weeks at −20°C.

### Cell culture medium

**Table:** 

Cell culture medium	Amount (ml)	Final concentration
Dulbecco's Modified Eagle Medium (DMEM)	445	
Heat‐inactivated fetal bovine serum (FBS)	50	10% (v/v)
10,000 U/ml penicillin‐streptomycin	5	100 U/ml

Store DMEM (Corning, cat. no. 10013CV) at 4°C per manufacturer's guidelines. Store heat‐inactivated FBS (Thermo Fisher, cat. no. A3840001) and penicillin‐streptomycin (Thermo Fisher, cat. no.15140122) at −20°C per manufacturer's guidelines. Thaw heat‐inactivated FBS and penicillin‐streptomycin before usage. Prepare the cell culture medium in a biosafety cabinet. Store the cell culture medium up to 12 months at 4°C.

### Cytoskeleton extraction buffer

**Table:** 

Cytoskeleton extraction buffer	Stock solution concentration	Amount (ml)	Final concentration
Triton X‐100	5% (w/v)	4	0.5% (w/v)
1,4‐Piperazinediethanesulfonic acid (PIPES)	1 M (pH 7.0)	4	0.1 M
Ethylene glycol‐bis(2‐aminoethylether)‐*N,N,N′,N′*‐tetraacetic acid (EGTA)	10 mM	4	1 mM
Magnesium chloride (Thermo Fisher, cat. no. AM9530G)	1 M	0.04	1 mM
Water		27.96	
Total		40	

Prepare 20 ml of 5% (w/v) Triton X‐100 by dissolving 1 g Triton X‐100 (Sigma Aldrich, cat. no. T9284‐100ML) in 19 ml water. Prepare 20 ml of 1 M PIPES buffer by dissolving 6 g PIPES (Sigma Aldrich, cat. no. P6757) in 15 ml of water, adjusting pH to 7.0 with 5 M sodium hydroxide solution, and adding water to a total volume of 20 ml. Prepare 20 ml of 10 mM EGTA solution by dissolving 76 mg EGTA (Sigma Aldrich, cat. no. 03777) in 20 ml water. Store the Triton X‐100, PIPES, and EGTA stock solutions and the prepared cytoskeleton extraction buffer up to 6 months at room temperature.

### Digestion buffer

**Table:** 

Digestion buffer	Stock solution concentration	Amount (ml)	Final concentration (/100 ml solution)
Triton X‐100	5% (w/v)	10	0.50 g
Ethylenediaminetetraacetic acid (EDTA; Thermo Fisher, cat. no. 15575020)	0.5 M, pH 8.0	0.2	0.2 ml (1 mM)
Tris(hydroxymethyl)aminomethane (Tris; Thermo Fisher, cat. no. AM9855G)	1 M, pH 8.0	5	5 ml (50 mM)
Sodium chloride	5 M	20	4.67 g (1 M)
Water		64.8	
Total		100	

Store digestion buffer up to 2 weeks at room temperature. For long‐term storage, store 10‐ml aliquots of digestion buffer up to 12 months at −20°C.

### Microtubule fixation solution

**Table:** 

Microtubule fixation solution	Amount (ml)	Final concentration
16% (w/v) paraformaldehyde (Electron Microscopy Sciences, cat. no. 15710)	1.5	3% (w/v)
8% (w/v) glutaraldehyde (Electron Microscopy Sciences, cat. no. 16019)	0.1	0.1% (w/v)
10× PBS (Corning, cat. no. 21040CV))	0.8	1×
Water	5.6	
Total	8	

Prepare microtubule fixation solution in a chemical fume hood. Use microtubule fixation solution when fresh, fixing cells immediately after making the solution.

### Monomer solution (colloquially known as Stock X)

**Table:** 

Monomer solution (“Stock X”)	Stock solution concentration (g/100 ml solution)	Amount (ml)	Final concentration after addition of APS and TEMED stock solutions (g/100 ml solution)
Sodium acrylate (see vendors below)	38 (33% (w/w) in water)	2.25	8.6
Acrylamide (Sigma Aldrich, cat. no. A9099‐25G)	50	0.5	2.5
*N,N*′‐Methylenebisacrylamide (Sigma Aldrich, cat. no. 146072‐100G)	2	0.75	0.15
Sodium chloride (Thermo Fisher, cat. no. AM9759)	29.2 (5 M)	4	11.7 (2 M)
PBS	10×	1	1×
Water		1.3	
Total		9.8	

It takes time to fully dissolve sodium acrylate, acrylamide, and *N,N*′‐methylenebisacrylamide into water. Vortexing and sonication can help to speed up this process, but do not heat the solutions, because monomers may prematurely gel into polymer form at higher temperatures. Store the acrylamide and *N,N*′‐methylenebisacrylamide stock solutions up to 6 months at 4°C. The sodium acrylate stock solution must be used immediately after making. The Stock X solution can be divided into aliquots of 980 µl each and stored for at least a month at −20°C. *Note*: Sodium acrylate batches from different vendors, or from different lots, can vary in quality. Low‐quality sodium acrylate may not completely dissolve in water at the relatively high concentration of 33% (w/w), or may appear yellow or orange when dissolved in water. If the solution is cloudy, or appears yellow or orange, discard the solution and switch to a new bottle of sodium acrylate. Clear or slightly yellow solutions of sodium acrylate can be used in ExM experiments. We have recently tried out sodium acrylate from the vendors below, but we always recommend checking the quality through the aforementioned checks, because quality can vary over time, even from a given vendor.

**Table:** 

Sodium acrylate vendor	Cat. no.
Combi‐Blocks	QC‐1489
AK Scientific	R624
BLDpharm	BD151354
Santa Cruz Biotechnology	sc‐236893B
Fisher Scientific	50‐750‐9773

### Quenching solution

**Table:** 

Quenching solution	Amount	Final concentration
Glycine (Sigma Aldrich, cat. no. G7126‐100G)	750 mg	100 mM
10× PBS	10 ml	1×
Water	90 ml	
Total	100 ml	

Store quenching solution up to 12 months at 4°C.

### Reduction solution

**Table:** 

Reduction solution	Amount	Final concentration
Sodium borohydride (Sigma Aldrich, cat. no. 213462‐25G)	10 mg	0.1% (w/v)
PBS	10 ml	1×
Total	10 ml	

The reduction solution must be made fresh before use. Store sodium borohydride at room temperature in a desiccated container. Sodium borohydride is reactive and should be handled with care. Weigh out 10 mg sodium borohydride into a 50‐ml tube, add 10 ml PBS, and pipet up and down ten times, using ∼5 ml of volume each time, to mix and dissolve the sodium borohydride. The dissolution of sodium borohydride in PBS will produce hydrogen gas bubbles; if there are no bubbles, the sodium borohydride solution has gone bad.

### TEMED stock solution

**Table:** 

TEMED stock solution	Amount	Stock solution concentration (g/100 ml solution)
TEMED (Thermo Fisher, cat. no. 17919)	1 g (1.29 ml)	10
Water	9 ml	
Total	10 ml	

Divide TEMED stock solution into 1‐ml aliquots and store at −20°C for up to 2 weeks.

## COMMENTARY

### Background Information

Optical microscopy is instrumental for studying biological structures in cells and tissues. Due to the diffraction of light by lenses, fluorophores within a few hundred nanometers (e.g., ∼300 nm) of each other are not resolvable when imaged with conventional, diffraction‐limited optical microscopes. Optical super‐resolution microscopy techniques (reviewed in Huang, Bates, & Zhuang, 2009) greatly improve the imaging resolution by separately detecting objects residing within a diffraction‐limited volume, so that their locations can be accurately determined; these techniques enable researchers to image many biological structures that are smaller than a few hundred nanometers, but they require specialized hardware.

Complementary to, but conceptually different from, optical super‐resolution microscopy techniques, we recently developed expansion microscopy (ExM, reviewed in Wassie et al., 2019) as a sample processing technique to physically enlarge preserved biological specimens. After ExM processing, biological objects within a diffraction‐limited volume are physically separated from one another and can thus be detected with conventional, diffraction‐limited microscopes. ExM realizes such physical separation by synthesizing a polyelectrolyte hydrogel throughout the sample, with biomolecules or their markers crosslinked to the hydrogel network. After softening the mechanical properties of the hydrogel‐embedded sample by protease digestion or biomolecule denaturation, the sample can be isotropically expanded via treatment with water.

ExM builds on discoveries going back several decades, including the study of the physics of polyelectrolyte hydrogels that swell vastly when immersed in water (Tanaka, Sun, Nishio, Swislow, & Shah, 1980), and the use of hydrogel embedding of biological specimens to improve staining and imaging (Hausen & Dreyer, 1981). In ExM, the hydrogel used is a swellable polyelectrolyte hydrogel, explicitly designed to be synthesized densely throughout a biological specimen (the polymer spacing may be as small as a few nanometers; Cohen, Ramon, Kopelman, & Mizrahi, 1992)); furthermore, the hydrogel is synthesized evenly throughout the sample, so that biomolecules that are crosslinked to the gel network can be separated isotropically in 3D, thus faithfully preserving their relative positions after expansion. In practice, the expansion process introduces errors of a few percent over length scales comparable to a microscope's field of view (Wassie et al., 2019), but these are negligible for the vast majority of biological questions currently being investigated, which are primarily concerned with the relative organization of molecules within cells and tissues.

We believe users can learn the principles of, and experimental skills for, the core processes of ExM through this exercise of visualizing immunostained microtubules in expanded HeLa cells. By practicing this protocol, users will get a head start on ExM and pave a smooth path toward using the evolving suite of ExM techniques (reviewed in Tillberg & Chen, 2019; Wassie et al., 2019; protocols in Asano et al., 2018, and posted at http://expansionmicroscopy.org) for their own applications.

### Troubleshooting

Potential problems and solutions are listed in Table 1.

**Table 1:** Troubleshooting the Protocol

Step	Potential problem	Solution
Steps 1‐10	Cells are dead (e.g., appearing as round, non‐adherent cells) or contaminated, or other problems associated with cell culture occur	Learn and practice aseptic techniques. Please refer to Phelan, (2007) for detailed instructions on aseptic techniques. We also found video resources, such as Cell Culture Basics (https://www.thermofisher.com/us/en/home/references/gibco‐cell‐culture‐basics.html), to be educational and useful.
Steps 11‐14	Immunostained microtubules, before expansion, show discontinuous and/or distorted morphologies in step 24	Distorted or discontinuous morphologies of microtubules suggest failed fixation steps. We suggest that the users use the fixative vendors recommended in this protocol. Always use fresh fixatives from the ampules provided by the manufacturer; these ampules are intended for one‐time use after opening. The microtubule fixation solution (see recipe) must be used freshly and cannot be stored. Measure the pH of the PBS solution to confirm that the value is ∼pH 7.4. If the above troubleshooting attempts fail, discard all solutions and purchase new ones from the recommended vendors.
Steps 11‐28	Liquid aspiration from a 24‐well plate damages cells on the coverslip	When aspirating liquid from a well, use one hand to hold the 24‐well plate and the other to hold the pipet. First push and hold the pipet plunger to dispense air out of the pipet tip so that the pipet is ready for drawing up liquid. Then, carefully move the pipet tip to the bottom edge of the well, and carefully tilt the plate ∼15° toward the pipet tip. Finally, slowly release the plunger to draw the liquid into the pipet tip. Do not aspirate the liquid directly from the cell layer, as that might damage the cells.
	Cells dry out between steps	To prevent drying of cells, we recommend that beginners simultaneously handle no more than two wells at a time, to minimize the time between liquid aspiration and addition steps.
Step 13	Immunostained microtubules, before expansion, show significant background fluorescence signals in step 24	The sodium borohydride reduction solution (see recipe), used to quench the fixatives and reduce background fluorescence, must be made fresh before use. Sodium borohydride, when fresh, should generate hydrogen gas bubbles when dissolved in PBS. If no hydrogen gas bubbles observed, use a new bottle of sodium borohydride.
Steps 15‐24	Immunostained microtubules, before expansion, show very weak immunostaining signals in step 24	Antibodies must be stored per manufacturer's instructions to ensure good quality. Secondary antibodies must be stored in the dark to prevent dye photobleaching. Use brightfield imaging to inspect the morphology of the cells to confirm successful fixation. If fixation is successful, the likely cause of weak immunostaining signals is a bad lot of antibodies. Replace both primary and secondary antibodies with newly purchased ones using the catalog numbers and vendors provided in this protocol.
Step 26	Immunostained microtubules show good imaging outcomes before expansion in step 24, but very weak or even no fluorescence signals after expansion in step 54	AcX, which crosslinks proteins to the hydrogel network, is an NHS ester and is prone to degradation by hydration. Therefore, AcX powder must be stored in a container with drying reagents (e.g., Drierite) at −20°C. AcX must be dissolved in anhydrous DMSO rather than regular DMSO, and the aliquoted AcX solution must be stored in a desiccating container at −20°C. Also, measure the pH of the PBS solution to confirm that the value is around pH 7.4, as high pH (> 9) will quickly hydrolyze AcX before it reacts with proteins. If the above troubleshooting attempts fail, discard all AcX solutions and purchase new materials from the recommended vendors.
Steps 29‐30	Gelation solution gels prematurely before step 37	The speed of polymerization significantly increases at higher temperature (e.g., at human body temperature of ∼37°C) or with high concentrations of APS and TEMED. To prevent premature gelation, first, all solutions (Stock X, APS, and TEMED) must be chilled on ice before mixing. APS, the radical polymerization initiator, must be added last. The gelation solution must be immediately placed back on ice after adding APS and vortexing. Keep the gelation solution on ice when transferring the solution to the 24‐well plate in step 30. The 24‐well plate must be kept on ice during the 5‐min incubation period in step 30. If the above instructions were strictly followed, but the gelation solution still prematurely gelled, it is possible that the concentration of the APS stock solution was mistakenly high. Make a fresh APS stock solution and redo the experiment.
Step 31	Coverslips or glass slides are broken during chamber construction	Use forceps to carefully pick up and discard broken coverslips or glass slides into a sharp waste container. Be very careful and avoid getting wounded by the broken glass. Then, redo step 31 with new glass slides and coverslips.
Steps 32‐34	Cell culture coverslip dries before the chamber is filled with gelation solution in step 35	Steps 33 and 34 must be finished within 2 min so that the cells will not dry out. If the user finds these steps to be demanding, we suggest practicing them with clean cell culture coverslips placed in a clean 24‐well plate before performing the actual protocol.
	Cell culture coverslip is broken	Use forceps to carefully pick up and discard broken coverslips into a biological sharp waste container. Be very careful and avoid getting wounded by the broken glass. Then, redo the experiments.
Step 35	Air bubbles are trapped in the gelation chamber	Use a pair of forceps to slowly lift one edge of the cell culture coverslip at a slight angle, until the air bubbles migrate to the edge of the coverslip and disperse, and then slowly place the cell culture coverslip back to rest on top of the two spacer stacks. Refill the chamber with gelation solution if necessary.
Step 40	Gel fails to form	Check the incubator and make sure the temperature is at 37°C. If the temperature is correct, the most likely next reason for failed gelation is bad reagents. Prepare new Stock X, APS, and TEMED solutions (ordering fresh reagents if needed) and redo the experiments.
Step 44	Gel remains attached to the coverslip or the Parafilm after ProK digestion	If the gel remains attached to the coverslip or Parafilm, it is most likely that the digestion step failed. Check the pH of the digestion buffer to make sure that it is ∼8.0. The ProK enzyme must be stored at −20°C. The gelled sample must be fully immersed in the ProK‐containing digestion buffer during the overnight digestion in step 43. If the above instructions were strictly followed, but the problem still persists, purchase fresh reagents and enzymes to make new ProK‐containing digestion solution.
	Gel cannot be located or is lost during removal of the digestion buffer	At this step, the gel is still relatively small and difficult to locate. First, we recommend illuminating the petri dish from different angles using a flashlight or a lamp to locate the gel. The gel will slightly scatter incident light and so should be visible when illuminated from different angles. Second, we recommend dispensing the aspirated digestion solution into another clean petri dish, rather than discarding it right away, so that if the gel is accidently drawn into the transfer pipet, it can possibly be recovered from the dispensed digestion solution. After the gel is secured, then discard the aspirated digestion solution appropriately.
Steps 44‐53	Gel is damaged	Always use a wet paint brush to handle the gel if needed. Be slow and careful when handling the gel. The most likely cause of gel damage is aspiration or solution addition in steps 46, 47, 48, and 51. During these steps, first locate the gel using the instructions listed above, and then slowly aspirate solution from around the gel, avoiding touching the gel itself. Tilt the petri dish to move liquids to the side of the dish opposite to the gel, so as to avoid potential contact with the gel when aspirating liquids. If only a small part of the gel is damaged, use a clean razor blade to cut off the damaged part, provided that the remaining part is big enough for downstream processing and imaging.
Steps 46‐48 and 51	Gel cannot be located	As the gel expands, it will be easier to locate. We recommend illuminating the petri dish from different angles using a flashlight or lamp to locate the gel. The gel will slightly scatter incident light and so should be visible when illuminated from different angles. Slightly tilt the petri dish at different angles to help with this process, as needed.
Step 53	Air bubbles are trapped between the bottom of the gel and the glass bottom of the 6‐well plate	Use a wetted paintbrush to gently press the top of the gel to expel large air bubbles. In the case of small air bubbles that were not removed, image regions of the sample that do not include these air bubbles during microscopy. Mounting smaller gels (e.g., ∼10 mm × 10 mm, which is still sufficiently large for imaging) is less likely to introduce air bubbles.
Other	Can I use this protocol for other types of specimens, for example, tissues?	No, this protocol is designed and optimized for visualizing immunostained microtubules in expanded HeLa cells. Please refer to other, more flexible and comprehensive protocols (Asano et al., 2018, and protocols posted at http://expansionmicroscopy.org) for imaging of other types of specimens.
	Can I store the expanded gel before mounting?	Yes, the expanded gel can be stored in water in the petri dish for up to 1 week at 4°C, in the dark to avoid photobleaching. For long‐term storage, remove the water from the petri dish containing the expanded gel, add 10 ml PBS solution, and store the petri dish for up to a few months at 4°C. The gel will shrink in PBS and can be re‐expanded, mounted, and imaged by first removing the PBS solution and then following steps 46‐54 of the protocol.
	Can I store the expanded and mounted gel?	Yes, the expanded and mounted gel can be stored for up to 1 week at 4°C in the dark.
	How should I properly dispose of used chemicals, gels, and solutions?	Follow instructions on the Material Safety Data Sheet (MSDS) provided by the manufacturers. Consult the Environment, Health and Safety (EHS) office of your institution for questions related to chemical and biological waste disposal.

### Understanding Results

When imaged under fluorescence microscopes, successfully fixed and immunostained HeLa cells will show bright, continuous, blurry lines indicating microtubule signals (Fig. 4A). The background fluorescence should be low.

Because cultured HeLa cells are transparent, hydrogel‐embedded samples will appear transparent throughout the entire ExM process. After gelation, the sample will look like a thin layer of transparent gel attached to the cell culture coverslip or, rarely, to the Parafilm (Fig. 3, part 1). After digestion with ProK, the sample will be detached from the coverslip or Parafilm and linearly expanded to ∼1.5 times its original size (Fig. 3, part 3). Then, after expansion with water, the gel will linearly expand to ∼4.5 times its original size.

When imaged under fluorescence microscopes, expanded HeLa cells will show bright, continuous, sharp lines representing microtubule signals (Fig. 4B). In expanded HeLa cells, immunostained microtubule filaments will be more visible, and resolved from one another, than those in HeLa cells before expansion (Fig. 4A) when imaged using the same microscope settings. Because microtubules are structured throughout the entire cell volume, one might estimate the size of the expanded HeLa cell by imaging a single *xy* plane at the bottom of the cell. The expanded HeLa cell should have a size of ∼100‐600 µm.
